# Use of Simulation to Improve Cardiopulmonary Resuscitation Performance and Code Team Communication for Pediatric Residents

**DOI:** 10.15766/mep_2374-8265.10555

**Published:** 2017-03-16

**Authors:** Kevin G. Couloures, Christine Allen

**Affiliations:** 1 Assistant Professor, Department of Pediatrics, Yale School of Medicine; 2 Assistant Professor, Department of Pediatrics, University of Oklahoma College of Medicine

**Keywords:** Simulation, Cardiopulmonary Resuscitation, Patient Handoff, Pediatrics, Handover, Pediatric Education, High Fidelity Simulation Training, Closed-Loop Communication, Teach-Back Communication

## Abstract

**Introduction:**

Cardiorespiratory events are infrequent in pediatric teaching hospitals but can lead to significant morbidity and mortality. Clear communication within the response team prevents delays in action and allows all team members to contribute to providing optimum management. This resource was developed to simulate high-acuity and low-frequency events for pediatric residents. The scenario options are recurrent supraventricular tachycardia, prolonged QT syndrome, myocarditis, and respiratory syncytial virus bronchiolitis.

**Methods:**

The simulation is best performed in a simulation center with audio- and video-recording capabilities but could also be performed in situ in the pediatric intensive care unit or emergency room. Necessary personnel include a simulation technician and two instructors. A code cart, mock medications, and defibrillator with hands-free pads appropriate for the mannequin are necessary supplies. Critical actions include initial survey and intervention, rhythm recognition, cardiopulmonary resuscitation (CPR), use of defibrillator, and administration of anti-arrhythmic medications when needed. At the conclusion of the scenario, a formal debriefing with learners using structured feedback is performed.

**Results:**

These cases have been used with groups of pediatric or emergency medicine residents approximately 16 times over the past 3 years. Learners have reported that participation increased their confidence and comfort with management of cardiorespiratory events and that communication technique practice improved their teamwork and sign-out skills. Rhythm recognition and CPR performance scores during the simulation scenarios improved, with subjective improvement during actual cardiorespiratory events.

**Discussion:**

This resource advances learner knowledge of Pediatric Advanced Life Support algorithms and teamwork communication and identifies learner knowledge and management deficits.

## Educational Objectives

By the end of this module, the learner will be able to:

1. Identify and begin treatment for a patient with cardiorespiratory compromise.
2. Initiate appropriate monitoring techniques.
3. Recognize cardiac arrhythmias and provide management applying the Pediatric Advanced Life Support algorithms.
4. Demonstrate proper use of the defibrillator to perform cardioversion and defibrillation.
5. Integrate closed-loop communication skills and early role assignment as ways to improve teamwork during resuscitation events.
6. Utilize SBAR (situation, background, assessment, and response) techniques to provide effective transitions of care.

## Introduction

Pediatric cardiorespiratory events are low-frequency but high-acuity events with high morbidity and mortality, both of which can be improved with early recognition and prompt resuscitation. In medical school or just prior to internship training, during Pediatric Advance Life Support (PALS) and Neonatal Advanced Life Support training courses,^1^ pediatric residents learn cardiopulmonary resuscitation (CPR) skills that are then reviewed annually or biannually. However, these skills deteriorate below satisfactory levels within just 6 months of training.^2^ Skill deterioration and the infrequent occurrence of cardiopulmonary arrest in pediatrics result in residents being inadequately prepared to resuscitate a child^3^ and experiencing high levels of anxiety during resuscitation events.^4^ van Schaik, Von Kohorn, and O'sullivan showed that residents’ confidence improved more with participation in mock codes than when they participated in actual resuscitation events. The greater confidence that resulted from participation in mock codes was attributed to the defined debriefing that occurs after a mock code.^5^

This resource was developed to simulate a high-acuity, low-frequency event. The target learners are pediatric residents, emergency department residents, pediatric intensive care unit fellows, pediatric emergency medicine fellows, and medical students. Participants must complete a PALS course prior to participation. This simulation is best performed in a simulation center with audio- and video-recording capabilities but can also be done in situ.

The primary goal of these simulation scenarios is to present four different disease processes—prolonged QT syndrome, myocarditis, bronchiolitis,^6^ or recurrent supraventricular tachycardia (SVT) syndrome^7^—that result in pediatric cardiorespiratory events and enable learners to quickly recognize and manage the inciting cause. In addition, learners engage in closed-loop communication techniques and use situation, background, assessment, and recommendation (SBAR) techniques to transfer the patient to a higher level of care. The combination of reviewing resuscitation and communication techniques results in the learner gaining skill and confidence in leading resuscitation events, which can be assessed through the use of a modified Clinical Performance Tool and surveys.

## Methods

These simulation cases were designed to offer learners an opportunity to use closed-loop communication, identify the underlying arrhythmia, provide a differential diagnosis, and practice hands-on management. We focused on clear role identification and communication to quickly diagnose the possible underlying etiology; use of PALS algorithms for bradycardia, SVT, asystole, ventricular fibrillation, and ventricular tachycardia; and use of the defibrillator.

The instructor should have familiarity with all four cases (Appendices A–D) and be part of the hospital code response team. Participants are sent a survey (Appendix G) at the beginning of the year, prior to the first scenario, to assess their previous experience and confidence in performing resuscitation. The simulation is best performed in a simulation lab with an area for debriefing but can also be performed in situ. An infant, young child, or adult simulation mannequin is needed for the exercise. The infant and young child mannequins are lying in bed at the start of the case, while the adult mannequin should be sitting up. The facilitator provides examples of closed-loop and SBAR communication techniques (Appendix E) for 5 minutes prior to the simulation exercise. The learners then enter the room and become familiar with the mannequin and monitoring capabilities (blood pressure cuff and EKG and pulse oximetry leads are attached) prior to the initiation of the scenario. The facilitator should read a short scenario with vital signs included. The mannequin has an antecubital IV line in place with a drain to a kidney basin. Other equipment needed is a defibrillator with appropriately sized hands-free pads, code cart with mock medications, and personal protective equipment. Code sheets, laboratory printouts, X-rays, and EKG printouts (Appendices J–N) for the scenario are available and should be given to the participants when requested.

Participants are divided into groups of four to five at the beginning of the scenario. Simulation personnel include the simulation technician to set up, program, and adjust the mannequin as necessary during the scenario. One faculty member stays in the control area and observes the action, acting as the consultant or parent if more information is needed. If the participant asks to call a parent or consultant, the faculty member answers any questions but remains unavailable to come to the room. A second faculty member stays in the room as a facilitator and observer and gives the team lab sheets and X-rays as requested.

The module takes 50 minutes to perform: 5 minutes of communication practice preceding the scenario, 25 minutes for the scenario, and 20 minutes to debrief. Directly following the resuscitation scenario, the learners meet in the debriefing room. The debriefing is led by a facilitator who reviews performance using the modified Clinical Performance Tool (Appendix F) and directs self-assessment and reflection from the participants using the questions provided (Appendix I). If areas of deficiency are identified, then the participants return to the simulation area to review proper technique. At the end of the year, the residents are sent a survey (Appendix H) to assess their experience and confidence when participating in real-life resuscitation.

### Equipment/Environment

#### Prolonged QT scenario

• Infant simulator on stretcher.
• Institution airway kit with the following supplies: laryngoscope with 0 and 1 blades, 3.0 and 3.5 endotracheal tubes cuffed and uncuffed with stylet. Bag valve mask for infant, infant face mask, infant nasal cannula, oral airway (60 and 70 mm), suction catheter and size 1 laryngeal mask airway.
• Defibrillator.
• Code cart with the following: IV supplies (22 g, 24 g, Fast IO), epinephrine, adenosine, midazolam, propofol, etomidate, ketamine, fentanyl, rocuronium, succinylcholine, lidocaine, amiodarone, calcium chloride, D10 glucose.
• Code sheet or similar for 5 kg and 8 kg, EKG, chest X-ray, and lab slips.
• Pulse oximetry and infant blood pressure cuff.

#### Myocarditis scenario

• Pediatric simulator on stretcher.
• Institution airway kit with the following supplies: laryngoscope with 1 and 2 blades, 4.5 and 5.0 endotracheal tubes cuffed and uncuffed with stylet. Bag valve mask for child, child face mask, pediatric nasal cannula, suction catheter, size 2 laryngeal mask airway, end-tidal carbon dioxide detector.
• Defibrillator.
• Code cart with the following: IV supplies (22 g, 24g, Fast IO), epinephrine, adenosine, midazolam, propofol, etomidate, ketamine, fentanyl, rocuronium, succinylcholine, lidocaine, amiodarone, calcium chloride, D10 glucose.
• Code sheet or similar for 25 kg and 30 kg, EKG, chest X-ray, and lab slips.
• Pulse oximetry and pediatric blood pressure cuff.

#### Recurrent SVT scenario

• Pediatric simulator on stretcher.
• Institution airway kit with the following supplies: laryngoscope with 1 and 2 blades, 4.5 and 5.0 endotracheal tubes cuffed and uncuffed with stylet. Bag valve mask for child, child face mask, pediatric nasal cannula, suction catheter, size 2 laryngeal mask airway, end-tidal carbon dioxide detector.
• Defibrillator.
• Code cart with the following: IV supplies (22 g, 24 g, Fast IO), epinephrine, adenosine, midazolam, propofol, etomidate, ketamine, fentanyl, rocuronium, succinylcholine, lidocaine, amiodarone, calcium chloride, D10 glucose.
• Code sheet or similar for 20 kg and 25 kg, EKG, chest X-ray, and lab slips.
• Pulse oximetry and pediatric blood pressure cuff.

#### Bronchiolitis scenario

• Infant simulator with warming table.
• Institution airway kit with the following supplies: laryngoscope with 0 and 1 blades, 3.0 and 3.5 endotracheal tubes cuffed and uncuffed with stylet. Bag valve mask for infant, neonatal face mask, infant nasal cannula and suction.
• Defibrillator.
• Code cart with the following: IV supplies (22 g, 24 g, Fast IO), epinephrine, adenosine, midazolam, propofol, etomidate, ketamine, fentanyl, rocuronium, succinylcholine, lidocaine, amiodarone, calcium chloride, D10 glucose.
• Code sheet or similar for 5 kg and 8 kg, EKG, chest X-ray, and lab slips.
• Pulse oximetry and infant blood pressure cuff.

### Assessment Tool

A two-part Clinical Performance Tool is used to score the resuscitation scenarios. Part 1 of the tool scores the team's initial assessment and resuscitation of the patient. The team's score is based on its assessment of airway, breathing, and circulation. Critical tasks assessed are ensuring the patient is on monitors, reporting vital signs, applying oxygen, ensuring IV or intraosseous access, administering fluid bolus, and initiating CPR. Part 2 of the tool evaluates the team's critical task performance for cardiac rhythm recognition, effectiveness of CPR, and appropriate dose and administration of epinephrine. The scoring tool was modified from the Clinical Performance Tool, which was previously shown to have interrater reliability of 0.63 and low variance (2.4%).^8^ In the interest of decreasing the length of the scenarios, we shortened the second part of the Clinical Performance Tool, which originally assessed learner performance of CPR three times. A similarly shortened scoring instrument for the PALS asystole scenario had an interrater reliability of 0.81 and low variance.^9^ In scenarios where the resident team correctly assesses the underlying etiology and begins effective treatment, the team is awarded the maximum score.

The communication skills assessment tool was constructed using similar logic to assess mastery of SBAR and closed-loop communication techniques. The first part of the tool assesses each component of the SBAR technique as not done (0 points), poor or incomplete (1 point), or complete (2 points). The second part of the tool assesses mastery of closed-loop communication techniques by evaluating whether a team leader was clearly identified, a person was clearly identified as responsible for carrying out actions, and orders were read back to the requestor. One scorer reviewed all our sessions and provided scoring.

## Results

These modules have been used with pediatric residents, emergency medicine residents, and pediatric emergency medicine fellows. Twenty-three pediatric residents completed the first-year trial of these four simulation sessions, which are now being used for both pediatric and emergency medicine residents. At study onset, 80% of residents reported that they had never been a resuscitation team leader, and none of them had led more than two resuscitations. At the end of the study year, 34% reported that they had been a resuscitation team leader. The nature of residents’ self-reported participation in resuscitation events changed during the year, with performance of bag valve mask ventilation, IV placement attempt, and defibrillation all increasing significantly by the end of the year. In addition, residents’ perception about being a team leader improved one full Likert-scale value, from *anxious* (1.2) to *somewhat anxious* (2.1), *p* < .0001. Similarly, their overall anxiety improved from *somewhat anxious* (2.1) to *neutral* (3.2), *p* < .001. Attending staff noted that the quality of resuscitations improved from *somewhat adequate* (2.2) to *good* (4.4), *p* = .05. We also found significant improvements using the modified Clinical Performance Tool to assess resident performance during the initial recognition phase (*p* = .05) and the subsequent arrhythmia assessment/CPR performance (*p* < .00001). All comparisons were performed and resultant *p* values calculated using the paired *t* test.

Communication scores also saw improvement. Scores from the SBAR component increased from an average score of 9 to 16 (*p* = .05), and the teamwork component average score increased from 5 to 9 (*p* = .05). The maximum score in the SBAR component is 18, and the maximum score in the teamwork component is 10. During follow-up interviews, the residents reported that the communication practice incorporated into the scenarios made a significant difference in their confidence, but we could not validate this result.

## Discussion

These cases were developed as an outgrowth of observed resident performance during actual resuscitation events, and the scenarios reflect the pathology that the residents encountered. The improvements seen in self-reported confidence, improved rhythm recognition, and resuscitation performance are consistent with earlier work demonstrating the utility of simulated cardiopulmonary arrest events to reduce anxiety and improve performance during actual events.^10^ Similar improvements have been demonstrated in the outpatient pediatric setting,^11^ where many of these residents will eventually practice. At our institution, residents have minimal participation in real-life code blue events. Only 12 events occurred in the inpatient units during the first year the scenarios were used. However, participants in our study performed active roles in mock code blue scenarios. These roles included serving as team leader and performing bag mask ventilation, CPR, and defibrillation. After performing these active roles, participants reported significant decreases in their anxiety and increased confidence in being the team leader. Earlier work showed that the integration of mock codes into a pediatric residency improved resident comfort, self-assessed skill proficiency, and survival rates.^12–14^

The incorporation of these scenarios into the curriculum addresses the deficit observed in a single-institution study of 34 mock code scenarios, which reported that pediatric residents deviated from American Heart Association (AHA) protocols 75% of the time and had communication errors 100% of the time.^15^ We were able to demonstrate that use of this curriculum significantly improved basic CPR skills, adherence to PALS algorithms, and recognition of cardiac arrhythmias. We attribute this improvement to the use of regular didactic sessions before each session and scripted debriefing sessions led by an AHA-certified PALS instructor. This is consistent with findings from the EXPRESS study, which showed that the use of scripted of debriefing improves knowledge acquisition and team leader performance.^16^

Our quarterly training approach did not place a significant strain on residents’ work duties. The current project directly addressed the deterioration of cardiopulmonary skills that has been observed as soon as 6 months after an intensive training session.^17^ Each quarterly session lasted under 1 hour, which caused minimal interruption in residents’ clinical duties, and was done in place of a scheduled morning or lunch-hour lecture.

Limitations include the small sample of self-selected residents and the fact that the initial performance and self-assessment data were collected over a single academic year at a single institution. A second limitation is that the residents’ levels of anxiety and confidence were self-reported in a survey that had not been previously validated. A third limitation is that the improvements in teamwork and care transitions were also self-reported. Finally, we were unable to demonstrate an impact upon patient outcomes since the hospital had already initiated the use of rapid response teams and the pediatric early warning score. (As a result of these measures, the overall number of cardiopulmonary arrests had already decreased by almost 50%.) These trends continued after the initiation of the quarterly training program, but we did not observe any further decline. A final limitation is that although these scenarios can be performed in situ, they are best suited for a fully staffed simulation lab with high-fidelity patient simulators.

## Appendices

A. Simulation Case 1.docx
B. Simulation Case 2.docx
C. Simulation Case 3.docx
D. Simulation Case 4.docx
E. Communication Techniques.docx
F. Modified Clinical Performance Tool.docx
G. Initial Self-Assessment Questionnaire.docx
H. Year-End Self-Assessment Questionnaire.docx
I. Debriefing Questions.docx
J. Simulation Scenario CBC.docx
K. Simulation Scenario EKG.docx
L. Simulation Scenario Images.pptx
M. Simulation Scenario iSTAT.docx
N. Simulation Scenario Lab Values.docx All appendices are peer reviewed as integral parts of the Original Publication.
