# Supplementary material for: Use of Simulation to Improve Cardiopulmonary Resuscitation Performance and Code Team Communication for Pediatric Residents
Source: MedEdPORTAL. 2017 Mar 16;13:10555. doi: 10.15766/mep_2374-8265.10555 (PMC6342167; doi:10.15766/mep_2374-8265.10555)
Supplement: Supplementary file 1 — A. Simulation Case 1.docx B. Simulation Case 2.docx C. Simulation Case 3.docx D. Simulation Case 4.docx E. Communication Techniques.docx F. Modified Clinical Performance Tool.docx G. Initial Self-Assessment Questionnaire.docx H. Year-End Self-Assessment Questionnaire.docx I. Debriefing Questions.docx J. Simulation Scenario CBC.docx K. Simulation Scenario EKG.docx L. Simulation Scenario Images.pptx M. Simulation Scenario iSTAT.docx N. Simulation Scenario Lab Values.docx [file mep-13-10555-s001.zip › A. Simulation Case 1.docx]

| **Appendix A: MedEdPORTAL Simulation Case Template**  **SIMULATION CASE TITLE: Prolonged QT in Pediatrics**  **AUTHORS: Kevin G. Couloures, DO, MPH – Yale University School of Medicine; Christine Allen, MD – University of Oklahoma School of Medicine** | |
| --- | --- |
| **PATIENT NAME: Bradley Branford**  **PATIENT AGE: 8 months old**  **CHIEF COMPLAINT: Acute Life Threatening Event** | |
|  | |
| **Brief narrative description of case**  *Include the presenting patient chief complaint and overall learner goals for this case* | 8-month old infant who has had a prior spell presents to the ED after a 20 second episode where he went limp but is now awake and crying vigorously. EMSA reports that when they first arrived on scene that the boy looked a little mottled but after supplying oxygen he cried vigorously and had stable vital signs. They did not perform any further interventions. He has now been placed into an exam room for further evaluation and becomes limp with poor perfusion.  Overall learner goals are to identify prolonged QT syndrome, understand and implement the PALS ventricular tachycardia with pulse and poor perfusion algorithm, and use of defibrillator to perform cardioversion |
| **Primary Learning Objectives**  *What should the learners gain in terms of knowledge and skill from this case? Use action verbs and utilize Bloom’s Taxonomy as a conceptual guide* | Primary Objectives   1. Recognize a patient with ventricular tachycardia and demonstrate the correct management for a patient with hemodynamic compromise as a result of ventricular tachycardia utilizing the AHA PALS VT algorithm 2. Utilize closed loop communication and SBAR techniques to work effectively with team members during the resuscitation.   Secondary Objectives   1. Formulate a differential diagnosis for a child with ventricular tachycardia and recite the initial steps to determine the underlying etiology. 2. Practice using the defibrillator for cardioversion with correct application of pads and choice of voltage. |
| **Critical Actions**  *List which steps the participants should take to successfully manage the simulated patient. These should be listed as concrete actions that are distinct from the overall learning objectives of the case.* | After the patient is connected to the monitor the learner should recognize Ventricular tachycardia with hemodynamic compromise. Learner should obtain further history. Laboratory work should be sent including BMP with magnesium and phosphorous, Blood Gas. Learner should ask for EKG.  Desired Action: Cardioversion, if performed will return to sinus rhythm for 3 minutes but then recur unless lidocaine or amiodarone are ordered.  If no cardioversion is performed then the infant will progress to asystole  Adenosine if ordered will not have any effect and patient will progress to asystole.  2^nd^ episode of ventricular tachycardia will progress to asystole unless lidocaine or amiodarone are administered again. |
| **Learner Preparation**  *What information should the learners be given prior to initiation of the case?* | PALS algorithm code cards  Brief review of proper closed loop communication techniques  Modeling of proper SBAR communication technique |

| Initial Presentation | | | |
| --- | --- | --- | --- |
| **Initial vital signs** | T -99, HR-138, RR- 28, sPO2 – 100%. Upon triage | | |
| **Overall Appearance**  *What do learners see when they first enter the room?* | General: Infant limp with weak cry while on the stretcher | | |
| **Actors and roles in the room at case start**  *Who is present at the beginning and what is their role? Who may play them?* | Facilitator: Supplies initial History, provides lab slips, EKG, and radiology results as requested  Faculty member in control room to assist with simulation. Provides additional history when asked. | | |
| **HPI**  *Please specify what info here and below must be asked vs what is volunteered by patient or other participants* | He is taken to a room and initially doing okay but then has another episode of becoming limp and the team has been called into the room. | | |
| **Past Medical/Surgical History** | **Medications** | **Allergies** | **Family History** |
|  | None | None | Paternal cousin who has “spells” |
| **Physical Examination** | | | |
| **General** | Infant is limp, lying in bed with occasional cry | | |
| **HEENT** | Atraumatic, Pupils 4 mm and reactive bilaterally. Ears without erythema or fluid levels. Pharynx without erythema | | |
| **Neck** | Supple, no masses or lymphadenopathy | | |
| **Lungs** | Clear to ausculatation bilaterally. No rales, wheeze, or rhonchi | | |
| **Cardiovascular** | Tachycardic with poor perfusion. Cap refill 3-4 seconds. No murmur or rub appreciable | | |
| **Abdomen** | Soft, non-distended, non-tender, no organomegaly | | |
| **Neurological** | Cries occasionally and not responsive | | |
| **Skin** | No rash | | |
| **GU** | Normal male genitalia | | |
| **Psychiatric** | Appropriate for age | | |

| Instructor Notes - Changes and CASE Branch Points  *This section should be a list with detailed description of each step than may happen during the case. If medications are given, what is the response? Do changes occur at certain time points? Should the nurse or other participant prompt the learners at given points? Should new actors or participants enter, and when? Are there specific things the patient will say or do at given times? There are a few examples given, but it is expected that most cases will have many more changes and potential branch points.* | | |
| --- | --- | --- |
| **Intervention / Time point** | **Change in Case** | **Additional Information** |
| *Beginning of case* | Respiratory rate is decreasing. If EKG or cardiac monitor is requested then it will demonstrate ventricular tachycardia | BP will be 50/20 and respiration rate will be 8 with declining sPO2. |
| *Supplemental oxygen given or IV bolus of saline* | No change in BP or tachycardia |  |
| *Adenosine is given for suspected supraventricular tachycardia* | No change. | Patient begins to progress to asystole |
| *Cardioversion performed for ventricular tachycardia.* | Heart rate 140 with BP 65/37 | If learner asks how perfusion appears then improved to cap refill of 3 seconds**.** |
| *Amiodarone or Lidocaine not given within 3 minutes after cardioversion* | Heart rate 180, BP 50/20 | If learner asks how perfusion appears 4-5 seconds |
| *Amiodarone or Lidocaine given* | Heart rate 120, BP 60/31 | Perfusion 2-3 seconds |

**Debriefing Questions**

The facilitator will ask the participants to critique their management of the patient

Potential questions or discussion points are detailed below.

| **Key Question** | **Points to Discuss** |
| --- | --- |
| What went well during the resuscitation? Would you change anything?  How would the change affect performance? | Arrhythmia recognition: Prolonged QT.  How do you correct QT interval for rate?  QT/√RR (Bazett Formula)  CPR Performance: Compressions should be about 4 cm in most infants, 5 cm in most children.  Keep at a rate of 100 compressions per minute.  Fully release chest but maintain contact  Pause no more than 10 seconds |
| Were you able to form an effective team?  What made the team effective?  If not then what were the barriers to the team working together? | Team dynamics – how did this affect performance?  Role assignments –were they static or fluid?  Was there a single leader? Did this affect the way the team interacted? |
| Did you communicate effectively with each other?  What would have made the communication better? | Were closed loop communication techniques used?  Was positive readback performed?  Was SBAR (Situation Background Assessment Response) used? |
| Recognition of the differential diagnosis for the scenario presented | Prolonged QT: May be genetic, drug-induced, or due to electrolyte disorders |
| What is the appropriate management for the scenario | Prolonged QT |
| How do you perform defibrillation | Pad placement  Appropriate energy selection  Increased Joules on Defibrillator  Charge  Clear bystanders  Delivery of electrical energy  Immediate resumption of CPR |

**Ideal Scenario Flow**

*Provide a detailed narrative description of the way this case should flow if participants perform in the ideal fashion.*

*The learners enter the room to find a patient with a weak cry and poor inspiratory effort. They immediately place the patient on bedside monitors and recognize that the patient is tachycardic with an abnormal rhythm. Supplemental oxygen is provided and an IV fluid bolus is ordered with no improvement. After completing a physical examination and obtaining an appropriate history, the providers ask for an EKG and note that the rhythm is ventricular tachycardia with hemodynamic compromise. They then connect the defibrillator pads and apply the correct amount of joules. The learners will then administer lidocaine or amiodarone to prevent recurrence of the arrhythmia. The providers then give a SBAR summary of the patient and arrange for patient admission to the medical ICU.*

**Anticipated Management Mistakes**

*Provide a list of management errors or difficulties that are commonly encountered when using this simulation case.*

1. *Difficulty with interpretation of Cardiac Rhythm: We found when using this case with pediatric residents that they were often unsure of whether the rhythm was supraventricular tachycardia or ventricular tachycardia with narrow complex.*
2. *Failure to recognize the need for cardioversion: Some of our learners did not immediately recognize that the patient required cardioversion, leading to delay in diagnosis. We found it helpful to have the administration of adenosine not have any effect with progression to asystole as a way to prompt the learners to reconsider their diagnosis.*
3. *Uncertainty about how to use the defibrillator: Many of our learners were unfamiliar with the use of the defibrillator. We specifically covered this during the orientation to the simulation center and created specific debriefing materials on the use of the defibrillator.*
