# Supplementary material for: Use of Simulation to Improve Cardiopulmonary Resuscitation Performance and Code Team Communication for Pediatric Residents
Source: MedEdPORTAL. 2017 Mar 16;13:10555. doi: 10.15766/mep_2374-8265.10555 (PMC6342167; doi:10.15766/mep_2374-8265.10555)
Supplement: Supplementary file 1 — A. Simulation Case 1.docx B. Simulation Case 2.docx C. Simulation Case 3.docx D. Simulation Case 4.docx E. Communication Techniques.docx F. Modified Clinical Performance Tool.docx G. Initial Self-Assessment Questionnaire.docx H. Year-End Self-Assessment Questionnaire.docx I. Debriefing Questions.docx J. Simulation Scenario CBC.docx K. Simulation Scenario EKG.docx L. Simulation Scenario Images.pptx M. Simulation Scenario iSTAT.docx N. Simulation Scenario Lab Values.docx [file mep-13-10555-s001.zip › B. Simulation Case 2.docx]

| **Appendix B: MedEdPORTAL Simulation Case Template**  **SIMULATION CASE TITLE: Myocarditis Induced Arrhythmia**  **AUTHORS:**  **Kevin G. Couloures, DO, MPH – Yale University School of Medicine**  **Christine Allen, MD – University of Oklahoma School of Medicine** | |
| --- | --- |
| **PATIENT NAME: Samuel Sonesta**  **PATIENT AGE: 6 year old**  **CHIEF COMPLAINT: Poor PO intake** | |
|  | |
| **Brief narrative description of case**  *Include the presenting patient chief complaint and overall learner goals for this case* | You are called for a rapid response to an inpatient room for a 6-year-old patient that was just admitted from an outside hospital for a 1-week history of increasing lethargy, vomiting, and decreased po intake. Mom reports that Sam has had really decreased energy for the past week and has been fussier than usual. He has had some low-grade fever. In the OSH he was given a 40 cc/kg NS bolus for dehydration, blood culture sent and he was given a dose of rocephin (75mg/kg). The nurses on the floor called a rapid response upon his arrival because he is having some respiratory distress with RR in the 40’s. He is pale, difficult to arouse and his mom reports that he was not like this 30 minutes ago in the ED. |
| **Primary Learning Objectives**  *What should the learners gain in terms of knowledge and skill from this case? Use action verbs and utilize Bloom’s Taxonomy as a conceptual guide* | Primary Objectives   1. Recognize a patient with ventricular tachycardia deteriorating to ventricular fibrillation and demonstrate the correct management for a patient utilizing the AHA PALS VT, VFib and PEA algorithms 2. Utilize closed loop communication and SBAR techniques to work effectively with team members during the resuscitation.   Secondary Objectives   1. Formulate a differential diagnosis for a child with ventricular fibrillation and recite the initial steps to determine the underlying etiology. 2. Practice using the defibrillator for defibrillation with the correct application of pads and choice of voltage. |
| **Critical Actions**  *List which steps the participants should take to successfully manage the simulated patient. These should be listed as concrete actions that are distinct from the overall learning objectives of the case.* | After 5 minutes despite previous interventions, the patient should develop VTACH with a pulse.  Resident should respond with cardioversion, if they do not perform cardioversion (25-50 joules) within 2 minutes should deteriorate to pulseless VFIB  All other responses to VTACH should deteriorate to pulseless VFIB  If cardioversion is performed correctly the pt should convert to sinus rhythm. HR 170-180 BP 90/40 for approximately 2 minutes then develop VFIB  With the onset of VFIB the resident should start CPR immediately including BVM and adequate chest compressions.  Should cardiovert with 25-50 joules. Within 2 minutes or pt deteriorates to PEA  If CPR is initiated with BVM, chest compressions and is cardioverted within 2 minutes then pt will convert to sinus rhythm HR 170-180 BP 90/40 for 2 minutes then develop VFIB again and despite all interventions will deteriorate to PEA within 60 seconds  If after 3 minutes of developing VFIB, they have not performed cardioversion then the patient will deteriorate to PEA.  Once the patient is in PEA the resident should continue BVM, potentially consider intubation, they should continue chest compressions and ask for a code dose of epi within 2 minutes of developing PEA, if epi dose is given within 2 minutes of PEA the patient will recover and be in sinus rhythm, HR 170 BP 90/40 resident should then check pulse and proceed with intubation  If epi dose is not given within 2 minutes the patient will continue in PEA until the 2^nd^ code dose of epi is given and then will return to sinus rhythm, HR 170, BP 90/40 resident should then check pulse and proceed with intubation  Resident must ask for appropriate ETT size, blade and intubation medications, verify tube placement with ETCO2, auscultate breath sounds and order chest x-ray. |
| **Learner Preparation**  *What information should the learners be given prior to initiation of the case?* | PALS algorithm code cards  Brief review of proper closed loop communication techniques  Modeling of proper SBAR communication technique |

| Initial Presentation | | | |
| --- | --- | --- | --- |
| **Initial vital signs** | VS temp 38.8, HR 180, RR 40-50’s Sats 95% BP 95/53 weight 25 Kg | | |
| **Overall Appearance**  *What do learners see when they first enter the room?* | He is sleepy arouses with stimulation and is able to answer questions appropriately, visibly tachypneic with some subcostal retractions  Lung crackles bilaterally  CV distant heart tones S1S2, murmur II-III/VI at LSB poor cap refill, equal but diminished pulses  Abdomen +hepatomegaly, otherwise negative. | | |
| **Actors and roles in the room at case start**  *Who is present at the beginning and what is their role? Who may play them?* | Facilitator: Supplies initial History, provides lab slips, EKG, and radiology results as requested  Faculty member in control room to assist with simulation. Provides additional history when asked. | | |
| **HPI**  *Please specify what info here and below must be asked vs what is volunteered by patient or other participants* | Given: 6-year-old patient that was just admitted from an outside hospital for a 1-week history of increasing lethargy, vomiting, and decreased po intake. In the OSH he was given a 40 cc/kg NS bolus for dehydration, blood culture sent and he was given a dose of ceftriaxone (75mg/kg).  If asked: Mom reports that Sam has had really decreased energy for the past week and has been fussier than usual. He has had some low-grade fever. His mom reports that he was not like this 30 minutes ago in the ED. | | |
| **Past Medical/Surgical History** | **Medications** | **Allergies** | **Family History** |
| Previously well | Acetaminophen | Shellfish | Father with hypertension |
| **Physical Examination** | | | |
| **General** | He is sleepy arouses with stimulation and is able to answer questions appropriately, | | |
| **HEENT** | Atraumatic, Pupils 4 mm and reactive bilaterally. Ears without erythema or fluid levels. Pharynx without erythema | | |
| **Neck** | Supple, no masses or lymphadenopathy | | |
| **Lungs** | Tachypneic with some subcostal retractions. Lung crackles bilaterally | | |
| **Cardiovascular** | CV distant heart tones S1S2, murmur II-III/VI at LSB poor cap refill, equal but diminished pulses | | |
| **Abdomen** | Abdomen +hepatomegaly, otherwise negative. | | |
| **Neurological** | Appropriate for age | | |
| **Skin** | No rash or but appears flushed | | |
| **GU** | Tanner 1 male | | |
| **Psychiatric** | Appropriate for age | | |

| Instructor Notes - Changes and CASE Branch Points  *This section should be a list with detailed description of each step than may happen during the case. If medications are given, what is the response? Do changes occur at certain time points? Should the nurse or other participant prompt the learners at given points? Should new actors or participants enter, and when? Are there specific things the patient will say or do at given times? There are a few examples given, but it is expected that most cases will have many more changes and potential branch points..* | | |
| --- | --- | --- |
| **Intervention / Time point** | **Change in Case** | **Additional Information** |
| *Beginning of case* | Perfusion appears compromised | BP will be 90/50 and respiration rate will be 20 with declining sPO2. |
| *Supplemental oxygen given or IV bolus of saline* | Persistent tachycardia with worsening sPO2 and BP now 80/40. |  |
| *5 minutes into scenario* | Patient rhythm changes to ventricular tachycardia. | Patient no longer responsive to questions. Cap refill 4-5 seconds |
| *Cardioversion performed for ventricular tachycardia.* | Heart rate 170 with BP 80/38 | If learner asks how perfusion appears then improved to cap refill of 3 seconds**.** |
| *If no cardioversion* | No pulse  Ventricular Fibrillation | If learner asks how perfusion appears 4-5 seconds |
| *2 minutes after cardioversion for ventricular tachycardia* | Ventricular Fibrillation | If learner asks how perfusion appears 4-5 seconds |
| *Cardioversion performed for ventricular fibrillation.* | Heart rate 180 with BP 90/40 | If learner asks how perfusion appears then improved to cap refill of 3 seconds**.** |
| *If no cardioversion* | No pulse  Pulseless Electrical Activity | If learner asks how perfusion appears 4-5 seconds |
| *2 minutes after cardioversion for ventricular fibrillation* | Ventricular Fibrillation | If learner asks how perfusion appears 4-5 seconds |
| *60 seconds after second cardioversion* | Pulseless Electrical Activity |  |
| *Epinephrine given and Bag Valve Mask ventilation performed* | HR 170 BP 90/40 resident should then check pulse and proceed with intubation |  |
| *Intubation* | Verify tube placement with ETCO2, auscultate breath sounds and order chest x-ray. |  |

**Debriefing Questions**

The facilitator will ask the participants to critique their management of the patient

Potential questions or discussion points are detailed below.

| **Key Question** | **Points to Discuss** |
| --- | --- |
| What went well during the resuscitation? Would you change anything?  How would the change affect performance? | Arrhythmia recognition,  CPR Performance: Compressions should be about 4 cm in most infants, 5 cm in most children.  Keep at a rate of 100 compressions per minute.  Fully release chest but maintain contact  Pause no more than 10 seconds |
| Were you able to form an effective team?  What made the team effective?  If not then what were the barriers to the team working together? | Team dynamics – how did this affect performance?  Role assignments –were they static or fluid?  Was there a single leader? Did this affect the way the team interacted? |
| Did you communicate effectively with each other?  What would have made the communication better? | Were closed loop communication techniques used?  Was positive readback performed?  Was SBAR (Situation Background Assessment Response) used? |
| Recognition of the differential diagnosis for the scenario presented | Myocarditis: can be due to viral infection, autoimmune disease, toxins and medications |
| What is the appropriate management for the scenario | Myocarditis: |
| How do you perform defibrillation | Pad placement  Appropriate energy selection  Increased Joules on Defibrillator  Charge  Clear bystanders  Delivery of electrical energy  Immediate resumption of CPR |
| When is epinephrine indicated | Delivery routes and appropriate concentrations  Dosage |
| What is the sequence for Rapid Sequence Intubation | Medications used  How to select appropriate laryngoscope blade and ET tube size |

**Ideal Scenario Flow**

*Provide a detailed narrative description of the way this case should flow if participants perform in the ideal fashion.*

*The learners enter the room to find a school age child who is difficult to arouse. They immediately place the patient on bedside monitors and recognize that the patient is tachycardic. Supplemental oxygen is provided and an IV fluid bolus is ordered with no improvement. After completing a physical examination and obtaining an appropriate history, the providers ask for an EKG and note that the rhythm is ventricular tachycardia with hemodynamic compromise. They then connect the defibrillator pads and apply the correct amount of joules. The learners will then respond to the development of ventricular fibrillation with repeat cardioversion and CPR. When the patient develops PEA despite their interventions they will give epinephrine and intubate the patient. The providers will then give an SBAR summary of the patient and arrange for patient admission to the Pediatric ICU.*

**Anticipated Management Mistakes**

*Provide a list of management errors or difficulties that are commonly encountered when using this simulation case.*

1. *Difficulty with interpretation of Cardiac Rhythm: We found when using this case with pediatric residents that they were often unsure of whether the rhythm was ventricular tachycardia or ventricular fibrillation.*
2. *Failure to recognize the need for cardioversion: Some of our learners did not immediately recognize that the patient required cardioversion, leading to delay in diagnosis. We found it helpful to have the facilitator emphasize that the hemodynamics were unstable to help prompt the learners.*
3. *Uncertainty about how to use the defibrillator: Many of our learners were unfamiliar with the use of the defibrillator. We specifically covered this during the orientation to the simulation center and created specific debriefing materials on the use of the defibrillator.*
4. *Uncertainty about when to intubate: Many of our learners were unfamiliar with rapid sequence intubation and became more concerned about which drugs they should use rather than focusing on airway management and CPR. We specifically covered this during the orientation to the simulation center and created specific debriefing materials on rapid sequence intubation.*
