# Supplementary material for: Use of Simulation to Improve Cardiopulmonary Resuscitation Performance and Code Team Communication for Pediatric Residents
Source: MedEdPORTAL. 2017 Mar 16;13:10555. doi: 10.15766/mep_2374-8265.10555 (PMC6342167; doi:10.15766/mep_2374-8265.10555)
Supplement: Supplementary file 1 — A. Simulation Case 1.docx B. Simulation Case 2.docx C. Simulation Case 3.docx D. Simulation Case 4.docx E. Communication Techniques.docx F. Modified Clinical Performance Tool.docx G. Initial Self-Assessment Questionnaire.docx H. Year-End Self-Assessment Questionnaire.docx I. Debriefing Questions.docx J. Simulation Scenario CBC.docx K. Simulation Scenario EKG.docx L. Simulation Scenario Images.pptx M. Simulation Scenario iSTAT.docx N. Simulation Scenario Lab Values.docx [file mep-13-10555-s001.zip › C. Simulation Case 3.docx]

| **Appendix C: MedEdPORTAL Simulation Case Template**  **SIMULATION CASE TITLE: Recurrent SVT in Pediatrics**  **AUTHORS:**  **Kevin G. Couloures, DO, MPH – Yale University School of Medicine**  **Christine Allen, MD – University of Oklahoma School of Medicine** | |
| --- | --- |
| **PATIENT NAME: Angela Agnacio**  **PATIENT AGE: 7 years old**  **CHIEF COMPLAINT: Possible Seizure** | |
|  | |
| **Brief narrative description of case**  *Include the presenting patient chief complaint and overall learner goals for this case* | 7-year old girl who has had a prior febrile seizure presents to the ED after a 20 second episode where she went limp but is now awake and looks dazed. EMSA reports that when they first arrived on scene that the girl looked a little mottled but after supplying oxygen he cried vigorously and had stable vital signs. They did not perform any further interventions. |
| **Primary Learning Objectives**  *What should the learners gain in terms of knowledge and skill from this case? Use action verbs and utilize Bloom’s Taxonomy as a conceptual guide* | Primary Objectives   1. Recognize a patient with supraventricular tachycardia deteriorating to ventricular fibrillation and demonstrate the correct management for a patient utilizing the AHA PALS VT and VFib algorithms 2. Utilize closed loop communication and SBAR techniques to work effectively with team members during the resuscitation.   Secondary Objectives   1. Formulate a differential diagnosis for a child with supraventricular tachycardia and recite the initial steps to determine the underlying etiology. 2. Practice using the defibrillator for cardioversion and defibrillation with the correct application of pads and choice of voltage. |
| **Critical Actions**  *List which steps the participants should take to successfully manage the simulated patient. These should be listed as concrete actions that are distinct from the overall learning objectives of the case.* | Recognize supraventricular tachycardia  Desired Action: Cardioversion, if performed will return to sinus rhythm for 3 minutes but then recur unless amiodarone is given.  EKG should be ordered during this time along with electrolytes.  If adenosine is given then patient will progress to asystole.  2^nd^ episode of SVT will then progress to ventricular fibrillation until cardioversion is repeated and epinephrine is given.  SVT will recur unless amiodarone is given |
| **Learner Preparation**  *What information should the learners be given prior to initiation of the case?* | PALS algorithm code cards  Brief review of proper closed loop communication techniques  Modeling of proper SBAR communication technique |

| Initial Presentation | | | |
| --- | --- | --- | --- |
| **Initial vital signs** | T -99, HR-138, RR- 28, sPO2 – 100%. | | |
| **Overall Appearance**  *What do learners see when they first enter the room?* | She is taken to a room and initially doing okay and then has another episode of becoming limp. During this episode if EKG leads are in place then SVT will be apparent. BP will be 65/30 and respiration rate will be 6 with declining sPO2. | | |
| **Actors and roles in the room at case start**  *Who is present at the beginning and what is their role? Who may play them?* | Facilitator: Supplies initial History, provides lab slips, EKG, and radiology results as requested  Faculty member in control room to assist with simulation. Provides additional history when asked. | | |
| **HPI**  *Please specify what info here and below must be asked vs what is volunteered by patient or other participants* | Given: 7 year old female presents to the ED after a 20 second episode where she went limp but is now awake and looks dazed. EMSA reports that when they first arrived on scene that the girl looked a little mottled but after supplying oxygen he cried vigorously and had stable vital signs. They did not perform any further interventions.  If asked: Prior febrile seizure, no work-up at 5-1/2 years old | | |
| **Past Medical/Surgical History** | **Medications** | **Allergies** | **Family History** |
| Febrile seizure 2 years ago | Acetaminophen | Peanuts | Mother with Asthma |
| **Physical Examination** | | | |
| **General** | School age child who is not responding to parents | | |
| **HEENT** | Atraumatic, Pupils 5 mm – reactive bilaterally. Tympanic membrane erythematous with air fluid level on right | | |
| **Neck** | Supple, no lymphadenopathy | | |
| **Lungs** | Clear to auscultation bilaterally | | |
| **Cardiovascular** | Tachycardia with no appreciable murmur. Cap Refill 3-4 seconds | | |
| **Abdomen** | Soft, no masses palpable | | |
| **Neurological** | Difficult to arouse | | |
| **Skin** | No rash or erythema | | |
| **GU** | Not examined but mother reports no abnormalities | | |
| **Psychiatric** | Appropriate for age prior to illness | | |

| Instructor Notes - Changes and CASE Branch Points  *This section should be a list with detailed description of each step than may happen during the case. If medications are given, what is the response? Do changes occur at certain time points? Should the nurse or other participant prompt the learners at given points? Should new actors or participants enter, and when? Are there specific things the patient will say or do at given times? There are a few examples given, but it is expected that most cases will have many more changes and potential branch points..* | | |
| --- | --- | --- |
| **Intervention / Time point** | **Change in Case** | **Additional Information** |
| *Beginning of case* | Respiratory rate is decreasing. If EKG or cardiac monitor is requested then it will demonstrate supraventricular tachycardia | BP will be 65/30 and respiration rate will be 8 with declining sPO2. |
| *Supplemental oxygen given or IV bolus of saline* | No change in BP or tachycardia |  |
| *Adenosine is given for suspected supraventricular tachycardia* | No change. | Tachycardia and hemodynamic compromise persist |
| *Cardioversion performed for supraventricular tachycardia with hemodynamic compromise.* | Heart rate 140 with BP 75/45 | If learner asks how perfusion appears then improved to cap refill of 3 seconds**.** |
| *2 minutes after cardioversion Supraventricular tachycardia recurs* | Rhythm progresses to ventricular fibrillation |  |
| *Defibrillation performed and epinephrine given for ventricular fibrillation* | Rhythm stabilizes to sinus rhythm after defibrillation |  |
| *2 minutes after 2^nd^ cardioversion.* | Supraventricular tachycardia recurs with unstable hemodynamics | 3^rd^ defibrillation and amiodarone should be given |

**Debriefing Questions**

The facilitator will ask the participants to critique their management of the patient

Potential questions or discussion points are detailed below.

| **Key Question** | **Points to Discuss** |
| --- | --- |
| What went well during the resuscitation? Would you change anything?  How would the change affect performance? | Arrhythmia recognition: SVT  How do you differentiate SVT from sinus tachycardia?  Usually narrow complex but may look like Ventricular Tachycardia if there are conduction defects.  CPR Performance: Compressions should be about 4 cm in most infants, 5 cm in most children.  Keep at a rate of 100 compressions per minute.  Fully release chest but maintain contact  Pause no more than 10 seconds |
| Were you able to form an effective team?  What made the team effective?  If not then what were the barriers to the team working together? | Team dynamics – how did this affect performance?  Role assignments –were they static or fluid?  Was there a single leader? Did this affect the way the team interacted? |
| Did you communicate effectively with each other?  What would have made the communication better? | Were closed loop communication techniques used?  Was positive readback performed?  Was SBAR (Situation Background Assessment Response) used? |
| Recognition of the differential diagnosis for the scenario presented | SVT: one of the most common arrhythmias of children. Often due to a congenital defect in the conduction system. Peak incidences – infancy, early school age and adolescence. |
| What is the appropriate management for the scenario | SVT: |
| How do you perform cardioversion/defibrillation | Pad placement  Appropriate energy selection  Increased Joules on Defibrillator  Charge  Clear bystanders  Delivery of electrical energy  Immediate resumption of CPR |

**Ideal Scenario Flow**

*Provide a detailed narrative description of the way this case should flow if participants perform in the ideal fashion.*

*The learners enter the room to find a school age child who is difficult to arouse. They immediately place the patient on bedside monitors and recognize that the patient is tachycardic. Supplemental oxygen is provided and an IV fluid bolus is ordered with no improvement. After completing a physical examination and obtaining an appropriate history, the providers ask for an EKG and note that the rhythm is supraventricular tachycardia with hemodynamic compromise. They then connect the defibrillator pads and apply the correct amount of joules. The learners will then respond to the development of ventricular fibrillation with repeat cardioversion and CPR. They will then give amiodarone to prevent SVT recurrence. The providers will then give an SBAR summary of the patient and arrange for patient admission to the Pediatric ICU.*

**Anticipated Management Mistakes**

*Provide a list of management errors or difficulties that are commonly encountered when using this simulation case.*

1. *Difficulty with interpretation of Cardiac Rhythm: We found when using this case with pediatric residents that they were often unsure of whether the rhythm was supraventricular tachycardia or ventricular tachycardia.*
2. *Failure to recognize the need for cardioversion: Some of our learners did not immediately recognize that the patient required cardioversion, leading to delay in diagnosis. Some of the learners wanted to try vagal maneuvers despite the hemodynamic instability. We found it helpful to have the facilitator emphasize that the hemodynamics were unstable to help prompt the learners.*
3. *Uncertainty about how to use the defibrillator: Many of our learners were unfamiliar with the use of the defibrillator. We specifically covered this during the orientation to the simulation center and created specific debriefing materials on the use of the defibrillator.*
