# Supplementary material for: Use of Simulation to Improve Cardiopulmonary Resuscitation Performance and Code Team Communication for Pediatric Residents
Source: MedEdPORTAL. 2017 Mar 16;13:10555. doi: 10.15766/mep_2374-8265.10555 (PMC6342167; doi:10.15766/mep_2374-8265.10555)
Supplement: Supplementary file 1 — A. Simulation Case 1.docx B. Simulation Case 2.docx C. Simulation Case 3.docx D. Simulation Case 4.docx E. Communication Techniques.docx F. Modified Clinical Performance Tool.docx G. Initial Self-Assessment Questionnaire.docx H. Year-End Self-Assessment Questionnaire.docx I. Debriefing Questions.docx J. Simulation Scenario CBC.docx K. Simulation Scenario EKG.docx L. Simulation Scenario Images.pptx M. Simulation Scenario iSTAT.docx N. Simulation Scenario Lab Values.docx [file mep-13-10555-s001.zip › D. Simulation Case 4.docx]

| **Appendix D: MedEdPORTAL Simulation Case Template**  **SIMULATION CASE TITLE: Bronchiolitis**  **AUTHORS: Kevin G. Couloures, DO, MPH – Yale University School of Medicine; Christine Allen, MD – University of Oklahoma School of Medicine** | |
| --- | --- |
| **PATIENT NAME: Charlie Spears**  **PATIENT AGE: 3 weeks old**  **CHIEF COMPLAINT: Bronchiolitis** | |
|  | |
| **Brief narrative description of case**  *Include the presenting patient chief complaint and overall learner goals for this case* | The triage nurse comes running from the front with an infant in arms to the resuscitation bay and sets off the resuscitation alarm. The nurse states the baby was apneic and cyanotic when the mother rushed into the ED registration area yelling for help. |
| **Primary Learning Objectives**  *What should the learners gain in terms of knowledge and skill from this case? Use action verbs and utilize Bloom’s Taxonomy as a conceptual guide* | Primary Objectives   1. Recognize a patient with respiratory failure and demonstrate the correct management for a patient with hemodynamic compromise as a result of bradycardia utilizing the AHA PALS bradycardia algorithm 2. Utilize closed loop communication and SBAR techniques to work effectively with team members during the resuscitation.   Secondary Objectives   1. Formulate a differential diagnosis for a child with bradycardia/asystole and recite the initial steps to determine the underlying etiology. 2. Demonstrate knowledge of drugs used for Rapid Sequence Intubation |
| **Critical Actions**  *List which steps the participants should take to successfully manage the simulated patient. These should be listed as concrete actions that are distinct from the overall learning objectives of the case.* | - Recognizes respiratory failure - Recognizes imminent cardiac arrest - Performs primary survey and obtains all vital signs and AMPLE history - Establishes appropriate team roles - Recognizes airway/respiratory compromise - Performs bag-valve-mask ventilation - Establishes IV/IO access - Obtains bedside glucose/ Istat - Performs RSI using appropriate drugs, dosage, equipment size and technique. Confirms tube placement. Addresses post-intubation sedation needs - Seeks appropriate disposition and consultation - Communicates patient handoff effectively to accepting physician - Maintains appropriate communication with patient’s family |
| **Learner Preparation**  *What information should the learners be given prior to initiation of the case?* | PALS algorithm code cards  Brief review of proper closed loop communication techniques  Modeling of proper SBAR communication technique |

| Initial Presentation | | | |
| --- | --- | --- | --- |
| **Initial vital signs** | T -98, HR-90, RR- 10, sPO2 – 63%. | | |
| **Overall Appearance**  *What do learners see when they first enter the room?* | **Baby is apneic and cyanotic, HR 75, Pulse ox 63%, BP unavailable. WEIGHT 3KG, Temp 36.9** | | |
| **Actors and roles in the room at case start**  *Who is present at the beginning and what is their role? Who may play them?* | Facilitator: Supplies initial History, provides lab slips, EKG, and radiology results as requested  Faculty member in control room to assist with simulation. Provides additional history when asked. | | |
| **HPI**  *Please specify what info here and below must be asked vs what is volunteered by patient or other participants* | Given: 3-week-old infant with 2 day history of runny nose and cough.  If Asked: Refused last bottle and has not fed for 4 hours. Mother noted that he was breathing harder overnight. No fevers. No siblings. Aunt and cousins visited over weekend. | | |
| **Past Medical/Surgical History** | **Medications** | **Allergies** | **Family History** |
| 25-year-old mother delivered at 36 weeks.  Went home on DOL #3 | None | None Known | Mother has asthma – uses inhaler |
| **Physical Examination** | | | |
| **General** | Apneic and cyanotic | | |
| **HEENT** | Peri-oral cyanosis | | |
| **Neck** | Supple | | |
| **Lungs** | Fine rales throughout with poor inspiratory effort | | |
| **Cardiovascular** | Bradycardic for age with poor perfusion | | |
| **Abdomen** | Soft and nondistended, no masses palpable | | |
| **Neurological** | Unable to assess – not responsive | | |
| **Skin** | Cyanotic | | |
| **GU** | Normal for age – Tanner 1 | | |
| **Psychiatric** |  | | |

| Instructor Notes - Changes and CASE Branch Points  *This section should be a list with detailed description of each step than may happen during the case. If medications are given, what is the response? Do changes occur at certain time points? Should the nurse or other participant prompt the learners at given points? Should new actors or participants enter, and when? Are there specific things the patient will say or do at given times? There are a few examples given, but it is expected that most cases will have many more changes and potential branch points..* | | |
| --- | --- | --- |
| **Intervention / Time point** | **Change in Case** | **Additional Information** |
| *Beginning of case* | sPO2 continues to fall and heart rate declines to 62 |  |
| *Bag Valve Mask Ventilation is provided* | HR improves to 150, sPO2 increases to 93% |  |
| *If the team stops BVM or only supplies supplemental oxygen via facemask or nasal cannula* | sPO2 declines to 40% and heart rate declines to 40 | Team should recognize need for intubation at this time |
| *If team does not intubate the child or has not initiated BVM* | Asystole, sPO2 and HR without reading |  |
| *Rapid Sequence intubation is initiated and BVM is resumed* | Heart rate returns to 145 and sPO2 of 94% | Team should assess if IV is present during this time |
| During preparation for RSI | sPO2 declines to 70% | Patient has developed mucous plug which will be relieved with deep nasal suctioning |
| After RSI | sPO2 returns to 98% with HR of 160 |  |

**Debriefing Questions**

The facilitator will ask the participants to critique their management of the patient

Potential questions or discussion points are detailed below.

| **Key Question** | **Points to Discuss** |
| --- | --- |
| What went well during the resuscitation? Would you change anything?  How would the change affect performance? | Airway compromise: Increased posterior size of head and anterior airway put infant at higher risk of obstruction.  Continual reassessment – Helps to ensure that interventions are effective and if not that further measures are needed |
| Were you able to form an effective team?  What made the team effective?  If not then what were the barriers to the team working together? | Team dynamics – how did this affect performance?  Role assignments –were they static or fluid?  Was there a single leader? Did this affect the way the team interacted? |
| Did you communicate effectively with each other?  What would have made the communication better? | Were closed loop communication techniques used?  Was positive readback performed?  Was SBAR (Situation Background Assessment Response) used? |
| Recognition of the differential diagnosis for the scenario presented | Bronchiolitis: Describe how smaller airways, increased metabolic rate and lower physiologic reserve place infants at greater risk for respiratory failure |
| What is the appropriate management for the scenario | Bronchiolitis: |
| What are the appropriate medications for RSI | Propofol  Fentanyl  Midazolam  Vecuronium  Rocuronium  Succinyl Choline  Etomidate  Atropine |
| How do you determine the appropriate size laryngoscope blade and Endotracheal Tube size | 3-9 kg: 3.5 uncuffed, 3.0 Cuffed  Cuffed: age/4 +3  Uncuffed: age/4 + 4 |

**Ideal Scenario Flow**

*Provide a detailed narrative description of the way this case should flow if participants perform in the ideal fashion.*

*The learners enter the room to find an infant in severe respiratory distress. They immediately place the patient on bedside monitors and begin to apply positive pressure ventilation with good bag valve mask techniques. Once the patient has an improved pulse oximetry reading and heart rate they determine that supplemental oxygen is insufficient and that the patient will need to be intubated. They will confirm that the infant has a working IV and if it not working then a new one will be placed. During preparation for RSI they recognize that the decline in oxygen saturation is due to a mucous plug for which they perform deep nasal suctioning. Once the endotracheal tube has been placed the learners will confirm position via end-tidal CO2 and tape the tube in place. The providers will then give an SBAR summary of the patient and arrange for patient admission to the Pediatric ICU.*

**Anticipated Management Mistakes**

*Provide a list of management errors or difficulties that are commonly encountered when using this simulation case.*

1. *Hesitancy to begin Positive Pressure Ventilation: We found when using this case with pediatric residents that they often tried to place a nasal cannula or face mask for a patient in marked respiratory distress. In our debriefing we emphasized that rapid aggressive measures would prevent further deterioration.*
2. *Failure to recognize mucous plug as cause of desaturation: Some of our learners did not immediately recognize that the reason for desaturation during Bag Valve mask was not due to poor technique. We found it helpful to have the facilitator emphasize the DOPE mnemonic as a way to get the learners to focus on troubleshooting the entire process and not one aspect.*
3. *Uncertainty about when to intubate: Many of our learners were unfamiliar with rapid sequence intubation and became more concerned about which drugs they should use rather than focusing on airway management and CPR. We specifically covered this during the orientation to the simulation center and created specific debriefing materials on rapid sequence intubation.*
