# Supplementary material for: Use of Simulation to Improve Cardiopulmonary Resuscitation Performance and Code Team Communication for Pediatric Residents
Source: MedEdPORTAL. 2017 Mar 16;13:10555. doi: 10.15766/mep_2374-8265.10555 (PMC6342167; doi:10.15766/mep_2374-8265.10555)
Supplement: Supplementary file 1 — A. Simulation Case 1.docx B. Simulation Case 2.docx C. Simulation Case 3.docx D. Simulation Case 4.docx E. Communication Techniques.docx F. Modified Clinical Performance Tool.docx G. Initial Self-Assessment Questionnaire.docx H. Year-End Self-Assessment Questionnaire.docx I. Debriefing Questions.docx J. Simulation Scenario CBC.docx K. Simulation Scenario EKG.docx L. Simulation Scenario Images.pptx M. Simulation Scenario iSTAT.docx N. Simulation Scenario Lab Values.docx [file mep-13-10555-s001.zip › E. Communication Techniques.docx]

Appendix E: SBAR and Closed Communication Techniques

SBAR

- Situation
  - Identify yourself
  - Identify patient
  - Describe why calling
- Background
  - Patient’s presenting complaint
  - Relevant past medical history
  - Brief summary of code events
- Assessment
  - Current condition of patient
  - Current therapies
- Recommendation
  - Request for transfer to PICU
  - Clarifies request for additional studies
  - Indicates when transfer will occur.

Example:

S: Hello, this is John Jones, I am a 3^rd^ year Pediatrics Resident responding to a cardiorespiratory event for a 9 month old male – Bob Branford.

B: He is a 9 month old male who was brought to the ED for poor feeding and breathing fast. He responded well to suctioning and fluid bolus in the ED and was admitted to the pediatric unit last night. He had retractions, flaring and desaturation events this morning and a rapid response was initiated.

A: He has less tachypnea with oxygen saturation of 96% on 4 liters per minute of oxygen via nasal cannula. We have performed deep suctioning and trialed a dose of nebulized albuterol with no effect on respiratory effort.

P: Recommend transfer to PICU for increased respiratory support within the next hour.

Closed loop communication Pearls

Each team members identifies themselves and their role on the team.

Identify person that you are talking to by name (preferably) or role on team.

When asked to perform a task – repeat what you are going to do.

Example: I am checking the pulse and it is 140 bpm.

When a lab or study has been requested, ask how long that study will take and for progress reminders.
