# Supplementary material for: Use of Simulation to Improve Cardiopulmonary Resuscitation Performance and Code Team Communication for Pediatric Residents
Source: MedEdPORTAL. 2017 Mar 16;13:10555. doi: 10.15766/mep_2374-8265.10555 (PMC6342167; doi:10.15766/mep_2374-8265.10555)
Supplement: Supplementary file 1 — A. Simulation Case 1.docx B. Simulation Case 2.docx C. Simulation Case 3.docx D. Simulation Case 4.docx E. Communication Techniques.docx F. Modified Clinical Performance Tool.docx G. Initial Self-Assessment Questionnaire.docx H. Year-End Self-Assessment Questionnaire.docx I. Debriefing Questions.docx J. Simulation Scenario CBC.docx K. Simulation Scenario EKG.docx L. Simulation Scenario Images.pptx M. Simulation Scenario iSTAT.docx N. Simulation Scenario Lab Values.docx [file mep-13-10555-s001.zip › F. Modified Clinical Performance Tool.docx]

Appendix F: Modified Clinical Performance Tool

| **Task** | **O points** | **1 point** | **2 points**  *all components present* |
| --- | --- | --- | --- |
| Open Airway  Assess Patency | - Not Done | - > 30 seconds | - Opens Airway - Recognizes child is vocalizing - < 30 seconds |
| Assess Breathing | - Not Done | - > 30 seconds - Auscultation only, no recognition of decreased effort | - < 30 seconds, auscultation, tachypnea, and WOB |
| Oxygen | - Not Done | - Nasal Cannula or facemask | - 100% oxygen - via BVM or ETT |
| Monitors | - Not Done | - > 60 seconds - Pulse oximetry or cardiac monitors in place | - < 60 seconds - Pulse oximetry and cardiac monitor in place |
| Pulse Check | - Not Done | - > 30 seconds | - < 30 seconds |
| Blood Pressure | - Not Done | - >120 seconds | - <120 seconds |
| IV/IO | - Not Done | - PIV or IO placed in >120 seconds | - PIV or IO placed in < 120 seconds |
| Fluid Bolus  (Shock or Tachydysrhythmia) | - Not Done | - Wrong fluid ordered - Wrong amount | - 20 mL/kg isotonic fluid bolus ordered |
| CPR  (Asystole or Respiratory Arrest | - Not Done | - > 30 seconds after pulselessness - Poor technique | - <30 seconds after pulselessness - Good technique |
| **2^nd^ Stage** |  | | |
| Rhythm identification | - Not Done | - Does not verbalize rhythm, but demonstrates awareness | - Verbalizes correct rhythm |
| Effective Ventilation | - Not Done | - > 30 seconds after apnea occurs - Wrong rate | - < 30 seconds after apnea - Proper rate |
| CPR | - Not Done | - > 30 seconds after pulselessness - Poor technique (improper rate or depth) | - <30 seconds after pulselessness - Good technique (proper rate and depth) |
| Epinephrine | - Not given | - Incorrect dose - >30 seconds after asystole recognized - Suboptimal route | - Correct dose - < 30 seconds after asystole - Correct route (IV/IO) |
| Pulse Check | - Not Done | - > 60 seconds after change in status | - < 60 seconds after change in status |

**Note:** Four mock code scenarios were used – Prolonged QT, Myocarditis, Respiratory failure (Asystole), and Tachydysrhythmia (SVT). All of these scenarios will progress to asystole with return of spontaneous circulation after CPR is initiated and epinephrine is given.

**Reference:** Donoghue et al., Effect of High-Fidelity Simulation on Pediatric Advanced Life Support Training in Pediatric House Staff: A Randomized Trial. *Pediatric Emergency Care*. 2009.25(3):139-144.

Shortened from original version to only include 1^st^ administration of epinephrine and eliminates Return of Spontaneous Circulation check at the end.

**Communication Scoring Tool**

| **SBAR** | **O points** | **1 point** | **2 points** |
| --- | --- | --- | --- |
| **Situation** |  |  |  |
| Identify Yourself | - Not Done | - Partial or Poor Identification | - Well identified |
| Identify Patient | - Not Done | - Partial of Poor Identification | - Well identified |
| Describe why calling | - Not Done | - Vague or poor description | - Clear statement |
| **Background** |  |  |  |
| Patient’s Presenting Complaint | - Not Done | - Vague or poor description | - Clear and complete |
| Relevant PMH | - Not Done | - Vague or poor description | - Clear and complete |
| Brief code summary | - Not Done | - Vague or poor description | - Clear and complete |
| **Assessment** |  |  |  |
| Current Condition of Patient | - Not Done | - Vague or poor description | - Clear and complete |
| Current Therapies | - Not Done | - Vague or poor description | - Clear and complete |
| **Recommendations** |  |  |  |
| Transfer Request | - Not Done | - Vague or poorly worded request | - Clear and complete |
| **Teamwork** |  | | |
| **Leader Identified** |  |  |  |
| Leader initiated response | - Not Done | - Others started and then assumed role | - Initiated Response |
| Asked for team input | - Not Done | - Asked but did not recognize input | - Asked for input and acknowledged |
| **Positive Readback** |  |  |  |
| Orders stated clearly | - Not Done | - Orders unclear | - Clear |
| Person receiving orders clearly identified | - Not Done | - Personnel identification unclear | - Clear personnel identification |
| Readback | - Not Done | - Readback done with prompting | - Readback performed |
