# Supplementary material for: Use of Simulation to Improve Cardiopulmonary Resuscitation Performance and Code Team Communication for Pediatric Residents
Source: MedEdPORTAL. 2017 Mar 16;13:10555. doi: 10.15766/mep_2374-8265.10555 (PMC6342167; doi:10.15766/mep_2374-8265.10555)
Supplement: Supplementary file 1 — A. Simulation Case 1.docx B. Simulation Case 2.docx C. Simulation Case 3.docx D. Simulation Case 4.docx E. Communication Techniques.docx F. Modified Clinical Performance Tool.docx G. Initial Self-Assessment Questionnaire.docx H. Year-End Self-Assessment Questionnaire.docx I. Debriefing Questions.docx J. Simulation Scenario CBC.docx K. Simulation Scenario EKG.docx L. Simulation Scenario Images.pptx M. Simulation Scenario iSTAT.docx N. Simulation Scenario Lab Values.docx [file mep-13-10555-s001.zip › G. Initial Self-Assessment Questionnaire.docx]

Appendix G: Initial Resident Self-Assessment Questionnaire for Pediatric Resuscitation

1. What year of training are you in? PL1 PL2 PL3 PL4
2. How many pediatric resuscitations have you been to?

0 1-2 3-5 6-9 >9

1. For how many of these resuscitations have you stayed in the room until the patient was stabilized or transferred for further care?

0 1-2 3-5 6-9 >9

1. In how many of these resuscitations did you act as team leader?

0 1-2 3-5 6-9 >9

1. In how many of these resuscitations were you the first physician in the room?

0 1-2 3-5 6-9 >9

1. What tasks did you perform in the resuscitations that you have been to?

Please check all that apply

__Compressions __Bag Valve Mask Ventilation

__Intubation __IV Placement

__IO Placement __Defibrillation

1. What is your level of anxiety/confidence at participating in resuscitation?

(Very anxious) 1 2 3 (Neither) 4 5 (Very Confident)

1. What is your level of anxiety/confidence in running resuscitation as team leader?

(Very anxious) 1 2 3 (Neither) 4 5 (Very Confident)
