# Supplementary material for: Use of Simulation to Improve Cardiopulmonary Resuscitation Performance and Code Team Communication for Pediatric Residents
Source: MedEdPORTAL. 2017 Mar 16;13:10555. doi: 10.15766/mep_2374-8265.10555 (PMC6342167; doi:10.15766/mep_2374-8265.10555)
Supplement: Supplementary file 1 — A. Simulation Case 1.docx B. Simulation Case 2.docx C. Simulation Case 3.docx D. Simulation Case 4.docx E. Communication Techniques.docx F. Modified Clinical Performance Tool.docx G. Initial Self-Assessment Questionnaire.docx H. Year-End Self-Assessment Questionnaire.docx I. Debriefing Questions.docx J. Simulation Scenario CBC.docx K. Simulation Scenario EKG.docx L. Simulation Scenario Images.pptx M. Simulation Scenario iSTAT.docx N. Simulation Scenario Lab Values.docx [file mep-13-10555-s001.zip › H. Year-End Self-Assessment Questionnaire.docx]

Appendix I: Year-End Resident Self-Assessment Questionnaire for Pediatric Resuscitation

Resuscitation Survey

1. What year of training are you in? PL1 PL2 PL3 PL4
2. What is your level of anxiety/confidence at participating in resuscitation?

(Very anxious)1 2 3 (Neither) 4 5 (Very Confident)

1. What is your level of anxiety/confidence in running resuscitation as team leader?

(Very anxious)1 2 3 (Neither) 4 5 (Very Confident)

1. Did the use of closed loop communications skills improve your ability to work as a team member?

(Not Helpful)1 2 3 (Somewhat) 4 5 (Very Helpful)

1. Did the use of SBAR techniques improve your ability to provide sign-out during transitions of care?

(Not Helpful)1 2 3 (Somewhat) 4 5 (Very Helpful)

1. Did the Prolonged QT interval scenario help you recognize a child with ventricular tachycardia?

(Not Helpful)1 2 3 (Somewhat) 4 5 (Very Helpful)

1. Did the Myocarditis scenario allow you to recognize the difference between ventricular tachycardia and ventricular defibrillation?

(Not Helpful)1 2 3 (Somewhat) 4 5 (Very Helpful)

1. Did the Recurrent SVT scenario assist you in recognizing when a child requires cardioversion?

(Not Helpful)1 2 3 (Somewhat) 4 5 (Very Helpful)

1. Did the Bronchiolitis scenario adequately prepare you to recognize a child with respiratory failure?

(Not Helpful)1 2 3 (Somewhat) 4 5 (Very Helpful)
