# Supplementary material for: Use of Simulation to Improve Cardiopulmonary Resuscitation Performance and Code Team Communication for Pediatric Residents
Source: MedEdPORTAL. 2017 Mar 16;13:10555. doi: 10.15766/mep_2374-8265.10555 (PMC6342167; doi:10.15766/mep_2374-8265.10555)
Supplement: Supplementary file 1 — A. Simulation Case 1.docx B. Simulation Case 2.docx C. Simulation Case 3.docx D. Simulation Case 4.docx E. Communication Techniques.docx F. Modified Clinical Performance Tool.docx G. Initial Self-Assessment Questionnaire.docx H. Year-End Self-Assessment Questionnaire.docx I. Debriefing Questions.docx J. Simulation Scenario CBC.docx K. Simulation Scenario EKG.docx L. Simulation Scenario Images.pptx M. Simulation Scenario iSTAT.docx N. Simulation Scenario Lab Values.docx [file mep-13-10555-s001.zip › I. Debriefing Questions.docx]

Appendix I: Debriefing Questions

| What went well during the resuscitation? Would you change anything?  How would the change affect performance? | Arrhythmia recognition: Prolonged QT.  How do you correct QT interval for rate?  QT/√RR (Bazett Formula)  Arrhythmia recognition: SVT  How do you differentiate SVT from sinus tachycardia?  Usually narrow complex but may look like Ventricular Tachycardia if there are conduction defects.  Airway compromise: Increased posterior size of head and anterior airway put infant at higher risk of obstruction.  Continual reassessment – Helps to ensure that interventions are effective and if not that further measures are needed  CPR Performance: Compressions should be about 4 cm in most infants, 5 cm in most children.  Keep at a rate of 100 compressions per minute.  Fully release chest but maintain contact  Pause no more than 10 seconds |
| --- | --- |
| Were you able to form an effective team?  What made the team effective?  If not then what were the barriers to the team working together? | Team dynamics – how did this affect performance?  Role assignments –were they static or fluid?  Was there a single leader? Did this affect the way the team interacted? |
| Did you communicate effectively with each other?  What would have made the communication better? | Were closed loop communication techniques used?  Was positive readback performed?  Was SBAR (Situation Background Assessment Response) used? |
| Recognition of the differential diagnosis for the scenario presented | SVT: one of the most common arrhythmias of children. Often due to a congenital defect in the conduction system. Peak incidences – infancy, early school age and adolescence.  Prolonged QT: May be genetic, drug-induced, or due to electrolyte disorders  Myocarditis: can be due to viral infection, autoimmune disease, toxins and medications  Bronchiolitis: Describe how smaller airways, increased metabolic rate and lower physiologic reserve place infants at greater risk for respiratory failure |
| What is the appropriate management for the scenario | SVT:  Prolonged QT  Myocarditis:  Bronchiolitis: |
| How do you perform defibrillation | Pad placement  Appropriate energy selection  Increased Joules on Defibrillator  Charge  Clear bystanders  Delivery of electrical energy  Immediate resumption of CPR |
