# Supplementary material for: Use of Simulation to Improve Cardiopulmonary Resuscitation Performance and Code Team Communication for Pediatric Residents
Source: MedEdPORTAL. 2017 Mar 16;13:10555. doi: 10.15766/mep_2374-8265.10555 (PMC6342167; doi:10.15766/mep_2374-8265.10555)
Supplement: Supplementary file 1 — A. Simulation Case 1.docx B. Simulation Case 2.docx C. Simulation Case 3.docx D. Simulation Case 4.docx E. Communication Techniques.docx F. Modified Clinical Performance Tool.docx G. Initial Self-Assessment Questionnaire.docx H. Year-End Self-Assessment Questionnaire.docx I. Debriefing Questions.docx J. Simulation Scenario CBC.docx K. Simulation Scenario EKG.docx L. Simulation Scenario Images.pptx M. Simulation Scenario iSTAT.docx N. Simulation Scenario Lab Values.docx [file mep-13-10555-s001.zip › J. Simulation Scenario CBC.docx]

CBC – Prolonged QT

|  | Value | Reference Range |
| --- | --- | --- |
| WBC | 5 | *4.5-11* |
| Hgb | 11.7 | *12-16 gm/dL* |
| Hct | 35 | *36-46%* |
| MCV | 82 | *80-100 fl* |
| PLT | 245 | *150-400 th/cmm* |
| PMNs | 42 | *40-70%* |
| Lymph | 56 | *22-44%* |
| Eos | 2 | *0-8%* |

CBC – Myocarditis

|  | Value | Reference Range |
| --- | --- | --- |
| WBC | 9 | *4.5-11* |
| Hgb | 12.7 | *12-16 gm/dL* |
| Hct | 38 | *36-46%* |
| MCV | 88 | *80-100 fl* |
| PLT | 285 | *150-400 th/cmm* |
| PMNs | 54 | *40-70%* |
| Lymph | 42 | *22-44%* |
| Eos | 1 | *0-8%* |

CBC – Recurrent SVT

|  | Value | Reference Range |
| --- | --- | --- |
| WBC | 7 | *4.5-11* |
| Hgb | 12.9 | *12-16 gm/dL* |
| Hct | 39 | *36-46%* |
| MCV | 89 | *80-100 fl* |
| PLT | 310 | *150-400 th/cmm* |
| PMNs | 53 | *40-70%* |
| Lymph | 44 | *22-44%* |
| Eos | 2 | *0-8%* |

CBC – Bronchiolitis

|  | Value | Reference Range |
| --- | --- | --- |
| WBC | 12 | *4.5-11* |
| Hgb | 13 | *12-16 gm/dL* |
| Hct | 40 | *36-46%* |
| MCV | 82 | *80-100 fl* |
| PLT | 360 | *150-400 th/cmm* |
| PMNs | 39 | *40-70%* |
| Lymph | 60 | *22-44%* |
| Eos | 1 | *0-8%* |
