# Supplementary material for: Use of Simulation to Improve Cardiopulmonary Resuscitation Performance and Code Team Communication for Pediatric Residents
Source: MedEdPORTAL. 2017 Mar 16;13:10555. doi: 10.15766/mep_2374-8265.10555 (PMC6342167; doi:10.15766/mep_2374-8265.10555)
Supplement: Supplementary file 1 — A. Simulation Case 1.docx B. Simulation Case 2.docx C. Simulation Case 3.docx D. Simulation Case 4.docx E. Communication Techniques.docx F. Modified Clinical Performance Tool.docx G. Initial Self-Assessment Questionnaire.docx H. Year-End Self-Assessment Questionnaire.docx I. Debriefing Questions.docx J. Simulation Scenario CBC.docx K. Simulation Scenario EKG.docx L. Simulation Scenario Images.pptx M. Simulation Scenario iSTAT.docx N. Simulation Scenario Lab Values.docx [file mep-13-10555-s001.zip › M. Simulation Scenario iSTAT.docx]

Simulation Scenario Sample iSTAT values

iSTAT – Prolonged QT interval

|  | Value | Reference Range |
| --- | --- | --- |
| pH | 7.20 | *7.35-7.45* |
| pCO_2_ | 36 | *35-45* |
| pO_2_ | 120 | *80-120* |
| HCO_3_^-^ | 20 | *22-30 mMol/L* |
| Glucose | 95 | *60-110 mMol/L* |
| Na^+^ | 140 | *135-145 mMol/L* |
| K^+^ | 5.0 | *3.5-5.2 mMol/L* |
| Ionized Calcium | 1.1 | *1.1-1.3 mMol/L* |

iSTAT – Myocarditis

|  | Value | Reference Range |
| --- | --- | --- |
| pH | 7.21 | *7.35-7.45* |
| pCO_2_ | 52 | *35-45* |
| pO_2_ | 57 | *80-120* |
| HCO_3_^-^ | 20 | *22-30 mMol/L* |
| Glucose | 65 | *60-110 mMol/L* |
| Na^+^ | 143 | *135-145 mMol/L* |
| K^+^ | 5.4 | *3.5-5.2 mMol/L* |
| Ionized Calcium | 1.3 | *1.1-1.3 mMol/L* |

iSTAT – Recurrent SVT

|  | Value | Reference Range |
| --- | --- | --- |
| pH | 7.09 | *7.35-7.45* |
| pCO_2_ | 44 | *35-45* |
| pO_2_ | 65 | *80-120* |
| HCO_3_^-^ | 13 | *22-30 mMol/L* |
| Glucose | 95 | *60-110 mMol/L* |
| Na^+^ | 140 | *135-145 mMol/L* |
| K^+^ | 5.0 | *3.5-5.2 mMol/L* |
| Ionized Calcium | 1.2 | *1.1-1.3 mMol/L* |

iSTAT – Bronchiolitis

|  | Value | Reference Range |
| --- | --- | --- |
| pH | 7.10 | *7.35-7.45* |
| pCO_2_ | 60 | *35-45* |
| pO_2_ | 50 | *80-120* |
| HCO_3_^-^ | 18 | *22-30 mMol/L* |
| Glucose | 75 | *60-110 mMol/L* |
| Na^+^ | 138 | *135-145 mMol/L* |
| K^+^ | 5.2 | *3.5-5.2 mMol/L* |
| Ionized Calcium | 1.0 | *1.1-1.3 mMol/L* |
