# Supplementary material for: Use of Simulation to Improve Cardiopulmonary Resuscitation Performance and Code Team Communication for Pediatric Residents
Source: MedEdPORTAL. 2017 Mar 16;13:10555. doi: 10.15766/mep_2374-8265.10555 (PMC6342167; doi:10.15766/mep_2374-8265.10555)
Supplement: Supplementary file 1 — A. Simulation Case 1.docx B. Simulation Case 2.docx C. Simulation Case 3.docx D. Simulation Case 4.docx E. Communication Techniques.docx F. Modified Clinical Performance Tool.docx G. Initial Self-Assessment Questionnaire.docx H. Year-End Self-Assessment Questionnaire.docx I. Debriefing Questions.docx J. Simulation Scenario CBC.docx K. Simulation Scenario EKG.docx L. Simulation Scenario Images.pptx M. Simulation Scenario iSTAT.docx N. Simulation Scenario Lab Values.docx [file mep-13-10555-s001.zip › N. Simulation Scenario Lab Values.docx]

Laboratory Values – Prolonged QT

|  | Value | Reference Range |
| --- | --- | --- |
| Sodium (Na^+)^ | 141 | *135-145 mMol/L* |
| Potassium (K^+^) | 4.7 | *3.5-5.2 mMol/L* |
| Chloride (Cl^-^) | 101 | *95-107 mMol/L* |
| Bicarbonate (HCO_3_^-^) | 19 | *22-30 mMol/L* |
| BUN | 12 | *7-20 mMol/L* |
| Creatinine | 0.3 | *0.5-1.4 mMol/L* |
| Glucose | 65 | *60-110 mMol/L* |
| Magnesium | 1.9 | *1.7-2.2 mg/dL* |
| Ionized Calcium | 1.2 | *1.1-1.3 mMol/L* |

Laboratory Values – Myocarditis

|  | Value | Reference Range |
| --- | --- | --- |
| Sodium (Na^+)^ | 145 | *135-145 mMol/L* |
| Potassium (K^+^) | 4.9 | *3.5-5.2 mMol/L* |
| Chloride (Cl^-^) | 105 | *95-107 mMol/L* |
| Bicarbonate (HCO_3_^-^) | 14 | *22-30 mMol/L* |
| BUN | 20 | *7-20 mMol/L* |
| Creatinine | 0.6 | *0.5-1.4 mMol/L* |
| Glucose | 72 | *60-110 mMol/L* |
| Magnesium | 1.7 | *1.7-2.2 mg/dL* |
| Ionized Calcium | 1.1 | *1.1-1.3 mMol/L* |

Laboratory Values – Recurrent SVT

|  | Value | Reference Range |
| --- | --- | --- |
| Sodium (Na^+)^ | 141 | *135-145 mMol/L* |
| Potassium (K^+^) | 4.3 | *3.5-5.2 mMol/L* |
| Chloride (Cl^-^) | 102 | *95-107 mMol/L* |
| Bicarbonate (HCO_3_^-^) | 19 | *22-30 mMol/L* |
| BUN | 16 | *7-20 mMol/L* |
| Creatinine | 0.4 | *0.5-1.4 mMol/L* |
| Glucose | 83 | *60-110 mMol/L* |
| Magnesium | 1.8 | *1.7-2.2 mg/dL* |
| Ionized Calcium | 1.3 | *1.1-1.3 mMol/L* |

Laboratory Values – Bronchiolitis

|  | Value | Reference Range |
| --- | --- | --- |
| Sodium (Na^+)^ | 136 | *135-145 mMol/L* |
| Potassium (K^+^) | 4.9 | *3.5-5.2 mMol/L* |
| Chloride (Cl^-^) | 98 | *95-107 mMol/L* |
| Bicarbonate (HCO_3_^-^) | 19 | *22-30 mMol/L* |
| BUN | 8 | *7-20 mMol/L* |
| Creatinine | 0.2 | *0.5-1.4 mMol/L* |
| Glucose | 83 | *60-110 mMol/L* |
| Magnesium | 2.1 | *1.7-2.2 mg/dL* |
| Ionized Calcium | 1.2 | *1.1-1.3 mMol/L* |
